# Synthesis of Triphosphate Nucleoside Prodrugs: γ‐ProTriPs

**DOI:** 10.1002/cpz1.70291

**Published:** 2025-12-22

**Authors:** Camille Tisnerat, Fabrizio Pertusati, Michaela Serpi

**Affiliations:** ^1^ School of Chemistry Cardiff University Main Building, Park Place Cardiff Wales United Kingdom

**Keywords:** antiviral and anticancer prodrugs, nucleoside, nucleotide, triphosphate nucleotide prodrugs, γ‐ProTriP and microwave

## Abstract

Although monophosphate nucleoside prodrug approaches have been extensively investigated, leading to the development of several key antiviral and anticancer drugs, less attention has been given to the design of triphosphate prodrugs for the delivery of triphosphorylated nucleotide analogues. Expanding on this strategy, we report here an efficient synthetic methodology for preparing nucleoside triphosphate prodrugs, in which the γ‐phosphate of a nucleotide is masked with an amino acid ester and an aryloxy group (γ‐ProTriP). This approach aims to achieve the direct intracellular release of the triphosphate nucleotide active species, circumventing metabolic bottlenecks and potential toxicity that are often associated with the accumulation of nucleoside analogues and/or their mono‐ and diphosphate species. This article outlines the synthetic strategy for preparing γ‐ProTriP derivatives using either microwave‐accelerated synthesis or conventional heating methods. The approach is exemplified by the preparation of a clofarabine γ‐ProTriP, which emerges as a promising alternative to traditional monophosphate prodrug strategies. © 2025 The Author(s). *Current Protocols* published by Wiley Periodicals LLC.

**Basic Protocol**: Preparation of triphosphate aryloxy phosphoramidate of adenosine, uridine, and clofarabine with microwave heating

**Alternate Protocol**: Preparation of triphosphate aryloxy phosphoramidate of adenosine with conventional heating

**Support Protocol 1**: Cation exchange of UDP disodium salt to UDP di(triethylammonium) salt

**Support Protocol 2**: Synthesis of di(triethylammonium) salt of clofarabine 5′‐diphosphate

**Support Protocol 3**: Synthesis of pentafluorophenyl phosphorylating reagents

## Introduction

Nucleoside analogues (NAs) are a crucial class of compounds that form the basis of many antiviral and anticancer therapies currently available (Jordheim et al., 2013). After three sequential *in vivo* phosphorylations, NAs can inhibit viral and cellular DNA or RNA polymerases, leading to the disruption of viral replication and cancer cell proliferation. However, their therapeutic efficacy is often hindered by limited cellular uptake, metabolic instability, and slow speed and inefficiency of phosphorylation, which is necessary for their activation into bioactive triphosphate forms (Jordheim et al., 2013). To overcome the rate‐limiting first step of phosphorylation, various monophosphate prodrug strategies have been developed (Pradere et al., 2014), among which the ProTide approach (involving phosphate masked with an amino acid ester and an aryloxy group) proved to be the most successful, leading to three US Food and Drug Administration (FDA)‐approved antiviral drugs and several clinical candidates (Serpi & Pertusati, 2021).

However, certain NAs also suffer from slow or inefficient second and third phosphorylation steps (Albertioni et al., 1998; Bonate et al., 2006; Furman et al., 1986; Lavie et al., 1997; Xie & Plunkett, 1995) catalyzed by nucleoside monophosphate kinase (NMPK; Van Rompay et al., 2000) and nucleoside diphosphate kinase (NDPK; Bourdais et al., 1996), respectively. Despite their significant potential advantages, triphosphate prodrugs have received relatively little attention compared to monophosphate prodrugs, even though they could bypass the entire phosphorylation cascade and mitigate the metabolic challenges associated with the accumulation of the parent nucleoside or its mono‐ and diphosphate forms. To date, only a few studies have reported on the delivery of higher phosphorylated nucleosides (Bonnaffé et al., 1995; Bonnaffé et al., 1996; Hostetler et al., 1993; MacCoss et al., 1978; van Wijk et al., 1991).

As part of our ongoing efforts to develop novel NA prodrugs, we present here an efficient synthetic approach for the preparation of nucleoside triphosphate prodrugs (Tisnerat et al., 2025). This work describes a synthetic strategy for preparing novel triphosphate prodrugs in which the γ‐phosphate either of adenosine triphosphate (ATP) or uridine triphosphate (UTP) is masked with an aryloxy group and an amino acid ester (γ‐ProTriP), using either a microwave‐accelerated method or conventional heating. Support protocols for the synthesis of nucleoside diphosphate analogues and phosphorylating reagents are also included. Notably, we have successfully applied this synthetic strategy to clofarabine, an FDA‐approved purine nucleoside anticancer drug that suffers from a poor second phosphorylation step, offering a promising alternative to existing nucleotide prodrug approaches.

*CAUTION*: Carry out all operations involving organic solvents and reagents in a well‐ventilated fume hood. Wear appropriate protective clothing and eye/face protection.

*NOTE*: All reactions should be performed under anhydrous conditions.

*NOTE*: The ^31^P NMR spectra documented below were obtained with proton decoupling.

## PREPARATION OF TRIPHOSPHATE ARYLOXY PHOSPHORAMIDATES OF ADENOSINE, URIDINE, AND CLOFARABINE WITH MICROWAVE HEATING

**Figure 1:**
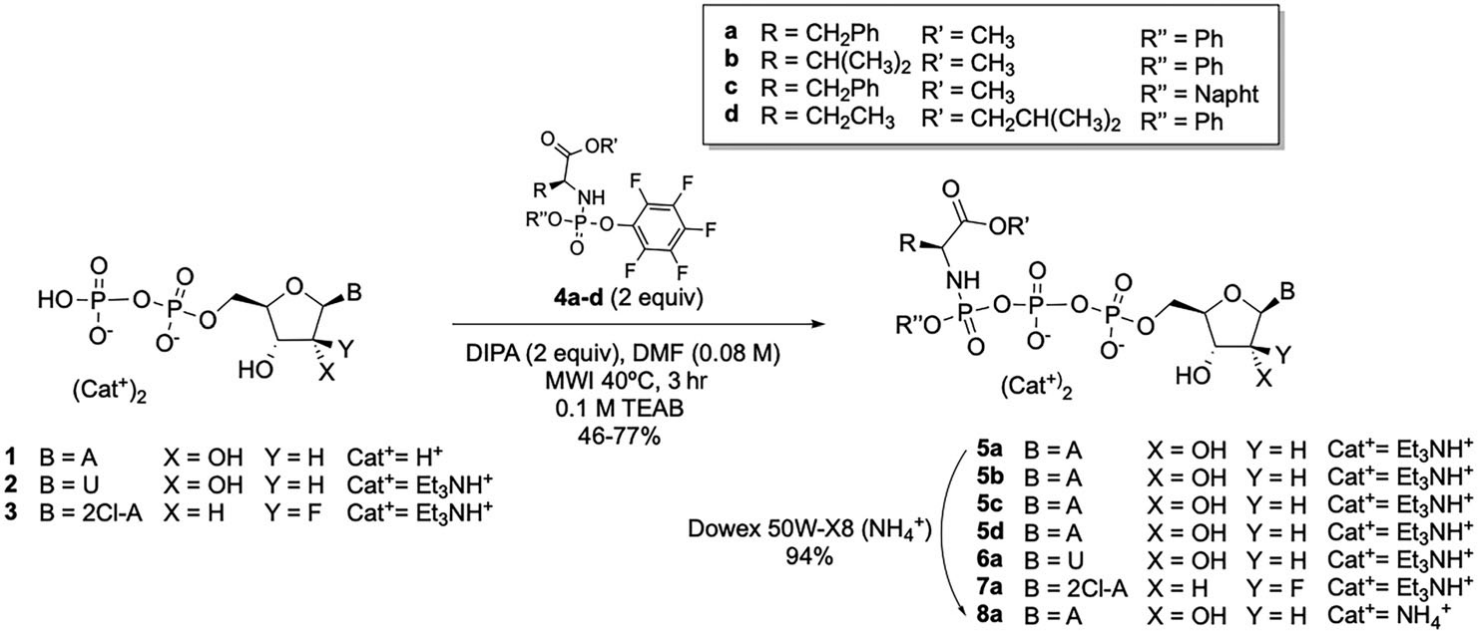
Synthesis of adenosine (**5a**‐**d**, **8a**), clofarabine (**7a**), and uridine (**6a**) γ‐ProTriPs with microwave irradiation (MWI).

This protocol describes the synthesis of purine‐ (**5a‐d**, **7a**, and **8a**) and pyrimidine‐based γ‐ProTriPs (**6a**) through reaction of the pentafluorophenyl phosphorylating reagents **4a**‐**d** with commercially available adenosine diphosphate (ADP, **1**), the di(triethylammonium) salt of uridine diphosphate (UDP, **2**), or the di(triethylammonium) salt of clofarabine 5′‐diphosphate (**3**) in the presence of diisopropylamine (DIPA) under microwave irradiation at 40°C for 3 hr (Fig. 1; Tisnerat et al., 2025). This phosphorylation method is faster than the conventional heating procedure described in the Alternate Protocol and allows a variety of pentafluorophenyl phosphorylating reagents to be used. As the physicochemical and biological properties of an active pharmaceutical ingredient (API) are affected by its salt form, this protocol also describes the exchange of the di(triethylammonium) cation in **5a** with an ammonium cation to afford the corresponding salt **8a** (steps 14‐23).

### Materials

Adenosine diphosphate (ADP) **1** (Carbosynth Ltd., BioSynth cat. no. NA10698), uridine diphosphate (UDP) di(triethylammonium) salt **2** (Support Protocol 1), *or* clofarabine di(triethylammonium) salt **3** (Support Protocol 2)
Pentafluorophenyl phosphorylating reagent **4a**, **4b**, **4c**, *or* **4d** (Support Protocol 3)
Anhydrous *N*,*N*‐dimethylformamide (DMF; Sigma‐Aldrich)
Anhydrous diisopropylamine (DIPA; Sigma‐Aldrich)
0.1 M triethylammonium bicarbonate (TEAB) buffer, pH 7.4 (see recipe)
Acetonitrile, HPLC grade (Sigma‐Aldrich)
28%‐30% ammonia solution (Sigma‐Aldrich)
Dowex 50W‐X8 (H^+^) resin (Sigma‐Aldrich)
Methanol (MeOH; Sigma‐Aldrich)
Deionized water
Triethylamine (Et_3_N; Sigma‐Aldrich)

2‐ to 5‐ml microwave vial sealed with aluminum cap fitted with a Teflon septum
Magnetic stirrer
Nitrogen
Schlenk line with vacuum pump and nitrogen trap
Microwave apparatus: Biotage Initiator+
Liquid nitrogen
10‐ and 100‐ml one‐neck round‐bottom flasks
Rotary evaporator equipped with vacuum pump and nitrogen trap
Sonication bath
Spatula
0.2‐µm PTFE filter
2‐ml syringe
Flash chromatography apparatus: Biotage Isolera One
Reverse‐phase column chromatography column: Biotage SNAP KP‐C18‐HS, 60‐g cartridge
UV light source
Nuclear magnetic resonance (NMR) instrument
Mass spectrometry instrument
High‐performance liquid chromatography (HPLC) system
10‐mm‐diameter glass column
Analytical thin‐layer chromatography plates: aluminum‐backed TLC plates, precoated with silica gel 60 F254, 0.2 mm (Merck Kieselgel)
10‐ml glass tubes

#### Perform phosphorylation reaction to obtain 5a‐d, 6a, and 7a

1 Suspend 50 mg (1 equiv, 0.12 mmol) **1**, 72 mg (1 equiv, 0.12 mmol) **2**, or 86 mg (1 equiv, 0.12 mmol) **3** in anhydrous DMF (1.5 ml) under a nitrogen atmosphere in a 2‐ to 5‐ml microwave vial containing a magnetic stirrer.
2 Add 120 mg (2 equiv, 0.24 mmol) **4a**, 109 mg (2 equiv, 0.24 mmol) **4b**, 115 mg (2 equiv, 0.24 mmol) **4c**, or 127 mg (2 equiv, 0.46 mmol) **4d**.
3 Add 34 µl (2 equiv, 0.24 mmol) DIPA.
4 Seal the vial with an aluminum cap fitted with a Teflon septum.
5 Stir and irradiate the reaction in a microwave reactor for 3 hr at 40°C with continuous irradiation power from 0 to 400 W, using the high absorbance level.
  A clear solution is obtained.
6 Transfer the solution into a 10‐ml one‐neck round‐bottom flask.
7 Evaporate the solvent using a rotary evaporator under reduced pressure with a nitrogen trap.
8 Dissolve the crude product in 1 ml of 0.1 M TEAB buffer and sonicate the resulting suspension to reduce particle size. If needed, scrape off the precipitate with a spatula and repeat sonication.
  Pentafluorophenol precipitates.
9 Carefully filter the suspension through a 0.2‐µm PTFE filter using a 2‐ml syringe.
10 Purify the crude product by automated flash chromatography with a Biotage Isolera One chromatographic system fitted with a Biotage SNAP KP‐C18‐HS (60‐g) column, using a solvent gradient from 95:5 (v/v) to 0:100 (v/v) 0.1 M TEAB buffer, pH 7.4/acetonitrile over 40 min at 50 ml/min flow rate. Monitor at two absorbance wavelengths, λ = 254 nm and 263 nm.
11 Combine the fractions containing the pure product and evaporate to dryness using a rotary evaporator under reduced pressure.
12 Dry the pure product under vacuum overnight to obtain **5a**‐**d**, **6a**, or **7a** (**5a** and **6a** as solids and **5b**‐**d** and **7a** as oils).
13 Characterize the compound by ^1^H, ^13^C, and ^31^P NMR, high‐resolution mass spectrometry (HRMS), and HPLC.

#### For 5a only: Perform cation exchange on 5a to afford salt 8a

14 Add Dowex 50W‐X8 (H^+^) resin to a 10‐mm‐diameter glass column to obtain a bed of 70 mm.
15 Wash the resin with methanol followed by deionized water (3 bed volumes each).
16 Equilibrate the resin with 28%‐30% ammonia solution in deionized water (3 bed volumes).
17 Wash the resin with deionized water until the pH reaches 7.0 (9 bed volumes).
18 Solubilize 55 mg of **5a** in 1 ml deionized water.
19 Load the **5a** solution onto the top of the column and drain into 10‐ml glass tubes.
20 Rinse the column with deionized water (3 bed volumes) and collect.
21 Spot all collected fractions on a TLC plate and combine the tubes containing the product (identified by their absorbance under UV light) into 100‐ml one‐neck round‐bottom flask.
22 Concentrate the solution using a rotary evaporator under reduced pressure and dry under vacuum overnight to obtain the diammonium salt **8a** as a glassy oil.
23 Characterize the compound by ^1^H, ^31^P, and ^13^C NMR and HRMS.
  Benzyl‐((((((((2R,3S,4R,5R)‐5‐(6‐amino‐9H‐purin‐9‐yl)‐3,4 dihydroxytetrahydrofuran‐2‐yl)methoxy)(hydroxy)phosphoryl)oxy)(hydroxy)phosphoryl)oxy)(phenoxy)phosphoryl)‐l‐alaninate di(triethylammonium) salt (**5a**). Yield of white solid 68%. ^1^H NMR (500 MHz, CD_3_OD) δ 8.54 (d, J = 1.8 Hz, 1H, H‐8), 8.19 (d, J = 1.5 Hz, 1H, H‐2), 7.35‐7.20 (m, 9H, Ar‐H), 7.13‐7.06 (m, 1H, Ar‐H), 6.08 (dd, J = 5.8, 4.6 Hz, 1H, H‐1′), 5.10‐5.06 (m, 2H, OCH_2_Ph), 4.69 (dd, J = 5.7, 5.3 Hz, 0.5H, H‐2′), 4.64 (dd, J = 5.4, 5.4 Hz, 0.5H, H‐2′), 4.51‐4.45 (m, 1H, H‐3′), 4.35‐4.19 (m, 3H, H‐4′, H‐5′), 4.21‐4.08 (m, 1H, CHCH_3_), 2.97 (q, J = 7.3 Hz, 12H, CH_3_CH_2_NH^+^), 1.45 (dd, J = 7.2, 0.9 Hz, 1.5H, CHCH_3_), 1.33 (dd, J = 7.1, 0.9 Hz, 1.5H, CHCH_3_), 1.20 (t, J = 7.3 Hz, 18H, CH_3_CH_2_NH^+^). ^31^P NMR (202 MHz, CD_3_OD) δ –7.52 (d, J = 18.2 Hz, 0.5P), –8.17 (d, J = 19.1 Hz, 0.5P), –11.74 (d, J = 20.7 Hz, 0.5P), –11.77 (d, J = 20.0 Hz, 0.5P), –23.81‐(‐25.02) (m, 1P). ^13^C NMR (126 MHz, CD_3_OD) δ 174.9 (d, J_CP_ = 6.1 Hz, C = O), 174.8 (d, J_CP_ = 7.1 Hz, C = O), 157.3 (C‐2), 153.82 (C‐6), 153.80 (C‐6), 152.4 (d, J_CP_ = 6.8 Hz, C‐Ar ipso OP), 152.3 (d, J_CP_ = 6.4 Hz, C‐Ar ipso OP), 151.0 (C‐4), 150.9 (C‐4), 141.13 (C‐8), 141.10 (C‐8), 137.40 (C‐Ar), 137.38 (C‐Ar), 130.52 (CH‐Ar), 130.52 (CH‐Ar), 130.49 (CH‐Ar), 130.48 (CH‐Ar), 129.5 (CH‐Ar), 129.2 (CH‐Ar), 125.81 (CH‐Ar), 125.79 (CH‐Ar), 125.74 (CH‐Ar), 125.73 (CH‐Ar), 121.95 (d, J_CP_ = 4.8 Hz, CH‐Ar), 121.91 (d, J_CP_ = 4.8 Hz, CH‐Ar), 120.17 (C‐5), 120.14 (C‐5), 88.9 (C‐1′), 88.8 (C‐1′), 85.7 (d, J_CP_ = 8.8 Hz, C‐4′), 85.6 (d, J_CP_ = 8.7 Hz, C‐4′), 76.13 (C‐2′), 76.09 (C‐2′), 72.16 (C‐3′), 71.96 (C‐3′), 67.73 (OCH_2_Ph), 67.69 (OCH_2_Ph), 66.8 (d, J_CP_ = 5.9 Hz, C‐5′), 66.7 (d, J_CP_ = 6.0 Hz, C‐5′), 51.9 (d, J_CP_ = 2.7 Hz, CHCH_3_), 51.6 (d, J_CP_ = 1.5 Hz, CHCH_3_), 47.31 (CH_3_CH_2_NH^+^), 20.81 (CHCH_3_), 20.76 (CHCH_3_), 9.74 (CH_3_CH_2_NH^+^). HRMS‐ESI (m/z): calcd. for C_26_H_32_N_6_O_14_P_3_ [M‐(Et_3_N)_2_+H]^+^ 745.1190, found 745.1189. HPLC reverse‐phase eluting with 0.1 M TEAB buffer, pH 7.4/CH_3_CN from 10/90 to 100/0 in 30 min, F = 1 ml/min, λ = 264 nm, t_R_ 8.507 min, purity 95%.
  Isopropyl ((((((((2R,3S,4R,5R)‐5‐(6‐amino‐9H‐purin‐9‐yl)‐3,4‐dihydroxytetrahydrofuran‐2‐yl)methoxy)(hydroxy)phosphoryl)oxy)(hydroxy)phosphoryl)oxy)(phenoxy)phosphoryl)‐l‐alaninate di(triethylammonium) salt (**5b**). Yield of colorless oil 62%. ^1^H NMR (500 MHz, CD_3_OD) δ 8.56 (s, 1H, H‐8), 8.20 (s, 1H, H‐2), 7.34‐7.25 (m, 9H, Ar‐H), 7.16‐7.06 (m, 1H, Ar‐H), 6.08 (dd, J = 5.7, 4.7 Hz, 1H, H‐1′), 4.97‐4.87 (m, 1H, OCH(CH_3_)_2_), 4.69 (dd, J = 5.5, 5.5 Hz, 0.5H, H‐2′), 4.65 (dd, J = 5.3, 5.3 Hz, 0.5H, H‐2′), 4.51‐4.44 (m, 1H, H‐3′), 4.34‐4.18 (m, 3H, H‐4′, H‐5′), 4.09‐3.97 (m, 1H, CHCH_3_), 3.16 (q, J = 7.3 Hz, 12H, CH_3_CH_2_NH^+^), 1.42 (dd, J = 7.1, 0.9 Hz, 1.5H, CHCH_3_), 1.31 (dd, J = 7.1, 0.8 Hz, 1.5H, CHCH_3_), 1.28 (t, J = 7.3 Hz, 18H, CH_3_CH_2_NH^+^), 1.22‐1.15 (m, 6H, OCH(CH_3_)_2_). ^31^P NMR (202 MHz, CD_3_OD) δ –7.48 (d, J = 18.2 Hz, 0.5P), –8.13 (d, J = 19.0 Hz, 0.5P), –11.75 (d, J = 20.4 Hz, 0.5P), –11.79 (d, J = 19.9 Hz, 0.5P), –24.26‐(‐24.61) (m, 1P). ^13^C NMR (126 MHz, CD_3_OD) δ 174.7 (d, J_CP_ = 6.6 Hz, C = O), 174.5 (d, J_CP_ = 7.7 Hz, C = O), 157.0 (C‐2), 153.51 (C‐6), 153.48 (C‐6), 152.4 (d, J_CP_ = 7.2 Hz, C‐Ar ipso OP), 152.3 (d, J_CP_ = 7.1 Hz, C‐Ar), 150.9 (C‐4), 150.8 (C‐4), 141.22 (C‐8), 141.20 (C‐8), 130.53 (CH‐Ar), 130.51 (CH‐Ar), 130.50 (CH‐Ar), 125.82 (CH‐Ar), 125.82 (CH‐Ar), 125.77 (CH‐Ar), 125.76 (CH‐Ar), 121.93 (d, J_CP_ = 4.8 Hz, CH‐Ar), 121.87 (d, J_CP_ = 4.9 Hz, CH‐Ar), 120.14 (C‐5), 88.9 (C‐1′), 88.8 (C‐1′), 85.65 (d, J_CP_ = 9.0 Hz, C‐4′), 85.58 (d, J_CP_ = 8.8 Hz, C‐4′), 76.15 (C‐2′), 76.11 (C‐2′), 72.1 (C‐3′), 71.9 (C‐3′), 69.92 (OCH(CH_3_)_2_), 69.87 (OCH(CH_3_)_2_), 66.8 (d, J_CP_ = 5.7 Hz, C‐5′), 66.7 (d, J_CP_ = 5.7 Hz, C‐5′), 51.9 (d, J_CP_ = 2.7 Hz, CHCH_3_), 51.6 (d, J_CP_ = 2.0 Hz, CHCH_3_), 47.4 (CH_3_CH_2_NH^+^), 22.0 (OCH(CH_3_)2), 21.95 (OCH(CH_3_)_2_), 21.88 (OCH(CH_3_)_2_), 20.91 (d, J_CP_ = 6.3 Hz, CHCH_3_), 20.87 (d, J_CP_ = 5.7 Hz, CHCH_3_), 9.1 (CH_3_CH_2_NH^+^). HRMS‐ESI (m/z): calcd. for C_22_H_32_N_6_O_14_P_3_ [M‐()_2_+H]^+^ 697.1189, found 697.1191. HPLC reverse‐phase eluting with 0.1 M TEAB buffer, pH 7.4/CH_3_CN from 10/90 to 100/0 in 30 min, F = 1 ml/min, λ = 254 nm, t_R_ 9.766 min, purity 93%.
  Ethyl ((((((((2R,3S,4R,5R)‐5‐(6‐amino‐9H‐purin‐9‐yl)‐3,4‐dihydroxytetrahydrofuran‐2‐yl)methoxy)(hydroxy)phosphoryl)oxy)(hydroxy)phosphoryl)oxy)(phenoxy)phosphoryl)‐l‐leucinate di(triethylammonium) salt (**5c**). Yield of colorless oil 69%. ^1^H NMR (500 MHz, CD_3_OD) δ 8.57 (s, 1H, H‐8), 8.21 (s, 1H, H‐2), 7.32‐7.24 (m, 4H, Ar‐H), 7.15‐7.07 (m, 1H, Ar‐H), 6.08 (dd, J = 5.7, 4.6 Hz, 1H, H‐1′), 4.68 (dd, J = 5.4, 5.4 Hz, 0.5H, H‐2′), 4.63 (dd, J = 5.3, 5.3 Hz, 0.5H, H‐2′), 4.51‐4.43 (m, 1H, H‐3′), 4.34‐4.20 (m, 3H, H‐4′, H‐5′), 4.15‐4.07 (m, 1H, OCH_2_CH_3_), 4.03 (q, J = 7.1 Hz, 1H, OCH_2_CH_3_), 3.97 (ddd, J = 9.8, 8.1, 6.7 Hz, 0.5H, CHCH_2_CH(CH_3_)_2_), 3.91 (ddd, J = 9.2, 7.9, 6.5 Hz, 0.5H, CHCH_2_CH(CH_3_)_2_), 3.16 (q, J = 7.3 Hz, 12H, CH_3_CH_2_NH^+^), 1.82‐1.73 (m, 0.5H, CHCH_2_CH(CH_3_)_2_), 1.65‐1.48 (m, 1.5H, CHCH_2_CH(CH_3_)_2_), 1.48‐1.43 (m, 1H, CHCH_2_CH(CH_3_)_2_), 1.28 (t, J = 7.3 Hz, 18H, CH_3_CH_2_NH^+^), 1.22 (t, J = 7.1 Hz, 1.5H, CH_2_CH_3_), 1.17 (t, J = 7.1 Hz, 1.5H, CH_2_CH_3_), 0.89 (dd, J = 6.6, 4.8 Hz, 3H, CHCH_2_CH(CH_3_)_2_), 0.81 (d, J = 6.4 Hz, 1.5H, CHCH_2_CH(CH_3_)_2_), 0.76 (d, J = 6.4 Hz, 1.5H, CHCH_2_CH(CH_3_)_2_). ^31^P NMR (202 MHz, CD_3_OD) δ –7.43 (d, J = 18.4 Hz, 0.5P), –7.57 (d, J = 18.5 Hz, 0.5P), –11.69 (d, J = 19.7 Hz, 0.5P), –11.74 (d, J = 19.6 Hz, 0.5P), –24.37 (d, J = 19.9 Hz, 0.5P), –24.52 (d, J = 19.6 Hz, 0.5P). ^13^C NMR (126 MHz, CD_3_OD) δ 175.0 (d, J_CP_ = 7.1 Hz, C = O), 174.9 (d, J_CP_ = 6.9 Hz, C = O), 156.62 (C‐6), 156.58 (C‐6), 152.95 (C‐2), 152.88 (C‐2), 152.45 (d, J_CP_ = 7.3 Hz, C‐Ar ipso OP), 152.41 (d, J_CP_ = 6.9 Hz, C‐Ar ipso OP), 150.82 (C‐4), 150.75 (C‐4), 141.4 (C‐8), 130.52 (CH‐Ar), 130.51 (CH‐Ar), 130.50 (CH‐Ar), 130.49 (CH‐Ar), 125.85 (CH‐Ar), 125.84 (CH‐Ar), 125.73 (CH‐Ar), 125.71 (CH‐Ar), 122.0 (d, J_CP_ = 4.8 Hz, CH‐Ar), 121.9 (d, J_CP_ = 4.9 Hz, CH‐Ar), 120.1 (C‐5), 89.00 (C‐1′), 88.9 (C‐1′), 85.64 (d, J_CP_ = 9.1 Hz, C‐4′), 85.60 (d, J_CP_ = 9.1 Hz, C‐4′), 76.22 (C‐2′), 76.16 (C‐2′), 72.1 (C‐3′), 71.9 (C‐3′), 66.72 (d, J_CP_ = 6.5 Hz, C‐5′), 66.67 (d, J_CP_ = 7.1 Hz, C‐5′), 62.1 (OCH_2_CH_3_), 62.0 (OCH_2_CH_3_), 54.63 (d, J_CP_ = 9.1 Hz, CHCH_2_CH(CH_3_)_2_), 54.62 (d, J_CP_ = 7.9 Hz, CHCH_2_CH(CH_3_)_2_), 47.4 (CH_3_CH_2_NH^+^), 44.6 (d, J_CP_ = 7.1 Hz, CHCH_2_CH(CH_3_)_2_), 44.4 (d, J_CP_ = 7.1 Hz, CHCH_2_CH(CH_3_)_2_), 25.5 (CHCH_2_CH(CH_3_)_2_), 25.4 (CHCH_2_CH(CH_3_)_2_), 23.02 (CHCH_2_CH(CH_3_)_2_), 22.97 (CHCH_2_CH(CH_3_)_2_), 22.5 (CHCH_2_CH(CH_3_)_2_), 22.3 (CHCH_2_CH(CH_3_)_2_), 14.5 (OCH_2_CH_3_), 14.4 (OCH_2_CH_3_), 9.1 (CH_3_CH_2_NH^+^). HRMS‐ESI (m/z): calcd. for C_24_H_36_N_6_O_14_P_3_ [M‐(Et_3_N)_2_+H]^+^ 725.1502, found 725.1508. HPLC reverse‐phase eluting with 0.1 M TEAB buffer, pH 7.4/CH_3_CN from 10/90 to 100/0 in 30 min, F = 1 ml/min, λ = 254 nm, t_R_ 11.120 min (fast‐eluting isomer) and 11.383 min (slow‐eluting isomer), purity 95%.
  Benzyl ((((((((2R,3S,4R,5R)‐5‐(6‐amino‐9H‐purin‐9‐yl)‐3,4‐dihydroxytetrahydrofuran‐2‐yl)methoxy)(hydroxy)phosphoryl)oxy)(hydroxy)phosphoryl)oxy)(naphthalen‐1‐yloxy)phosphoryl)‐l‐alaninate di(triethylammonium) salt (**5d**). Yield of colorless oil 46%. ^1^H NMR (500 MHz, CD_3_OD) δ 8.54 (d, J = 6.5 Hz, 1H, H‐8), 8.39‐8.26 (m, 1H, Ar‐H), 8.18 (d, J = 0.7 Hz, 1H, H‐2), 7.87‐7.78 (m, 1H, Ar‐H), 7.67‐7.56 (m, 2H, Ar‐H), 7.51‐7.43 (m, 2H, Ar‐H), 7.41‐7.31 (m, 1H, Ar‐H), 7.29‐7.16 (m, 5H, Ar‐H), 6.07 (dd, J = 5.4, 5.4 Hz, 1H, H‐1′), 5.02‐4.91 (m, 2H, OCH_2_Ph), 4.68 (dd, J = 5.4, 5.4 Hz, 0.5H, H‐2′), 4.64 (dd, J = 5.4, 5.4 Hz, 0.5H, H‐2′), 4.47 (ddd, J = 6.3, 5.0, 3.4 Hz, 1H, H‐3′), 4.36‐4.15 (m, 4H, H‐4′, H‐5′, CHCH_3_), 3.12 (q, J = 7.3 Hz, 12H, CH_3_CH_2_NH^+^), 1.44 (dd, J = 7.1, 1.0 Hz, 1.5H, CHCH_3_), 1.28 (dd, J = 7.1, 0.9 Hz, 1.5H, CHCH_3_), 1.24 (t, J = 7.3 Hz, 18H, CH_3_CH_2_NH^+^). ^31^P NMR (202 MHz, CD_3_OD) δ –7.21 (d, J = 18.1 Hz, 0.5P), –8.07 (d, J = 18.9 Hz, 0.5P), –11.70 (d, J = 20.7 Hz, 0.5P), –11.73 (d, J = 19.9 Hz, 0.5P), –24.16‐(‐24.58) (m, 1P). ^13^C NMR (126 MHz, CD_3_OD) δ 174.9 (d, J_CP_ = 6.1 Hz, C = O), 174.7 (d, J_CP_ = 6.5 Hz, C = O), 157.21 (C‐6), 157.19 (C‐6), 153.75 (C‐2), 150.91 (C‐4), 150.86 (C‐4), 148.7 (d, J_CP_ = 7.3 Hz, C‐Ar ipso OP), 148.2 (d, J_CP_ = 7.1 Hz, C‐Ar ipso OP), 141.12 (C‐8), 141.09 (C‐8), 137.25 (C‐Ar), 137.24 (C‐Ar), 136.15 (C‐Ar), 136.15 (C‐Ar), 129.46 (CH‐Ar), 129.45 (CH‐Ar), 129.42 (CH‐Ar), 129.11 (CH‐Ar), 129.07 (CH‐Ar), 129.06 (CH‐Ar), 128.57 (CH‐Ar), 128.53 (CH‐Ar), 128.12 (d, J_CP_ = 6.4 Hz, CH‐Ar), 128.08 (d, J_CP_ = 6.5 Hz, CH‐Ar), 127.63 (CH‐Ar), 127.58 (CH‐Ar), 127.35 (CH‐Ar), 127.29 (CH‐Ar), 126.60 (d, J_CP_ = 1.8 Hz, C‐Ar), 126.58 (d, J_CP_ = 1.9 Hz, C‐Ar), 125.61 (CH‐Ar), 125.54 (CH‐Ar), 123.54 (CH‐Ar), 120.17 (C‐5), 120.12 (C‐5), 116.50 (d, J_CP_ = 3.0 Hz, CH‐Ar), 116.46 (d, J_CP_ = 3.4 Hz, CH‐Ar), 88.93 (C‐1′), 88.84 (C‐1′), 85.60 (d, J_CP_ = 8.9 Hz, C‐4′), 85.55 (d, J_CP_ = 9.0 Hz, C‐4′), 76.08 (C‐2′), 72.15 (C‐3′), 71.97 (C‐3′), 67.68 (OCH_2_Ph), 67.65 (OCH_2_Ph), 66.81 (d, J_CP_ = 6.2 Hz, C‐5′), 66.76 (d, J_CP_ = 6.9 Hz, C‐5′), 52.02 (d, J_CP_ = 2.7 Hz, CHCH_3_), 51.66 (d, J_CP_ = 1.5 Hz, CHCH_3_), 47.43 (CH_3_CH_2_NH^+^), 20.85 (d, J_CP_ = 6.6 Hz, CHCH_3_), 20.80 (d, J_CP_ = 6.4 Hz, CHCH_3_), 9.10 (CH_3_CH_2_NH^+^). HRMS‐ESI (m/z): calcd/ for C_30_H_34_N_6_O_14_P_3_ [M‐(Et_3_N)_2_+H]^+^ 795.1346, found 795.1347. HPLC reverse‐phase eluting with 0.1 M TEAB buffer, pH 7.4/CH_3_CN from 10/90 to 100/0 in 30 min, F = 1 ml/min, λ = 254 nm, t_R_ 12.832 min (fast‐eluting isomer) and 12.936 min (slow‐eluting isomer), purity 90%. ^31^P‐NMR purity 99%.
  Benzyl ((S)‐(((((((2R,3S,4R,5R)‐5‐(2,4‐dioxo‐3,4‐dihydropyrimidin‐1(2H)‐yl)‐3,4‐dihydroxytetrahydro furan‐2‐yl)methoxy)(hydroxy)phosphoryl)oxy)(hydroxy)phosphoryl)oxy)(phenoxy)phosphoryl)‐l‐alaninate di(triethylammonium) salt (**6a**).Yield of a white solid 77%. ^1^H NMR (500 MHz, CD_3_OD) δ 8.04 (dd, J = 8.1, 6.0 Hz, 1H, H‐6), 7.37‐7.24 (m, 9H, Ar‐H), 7.17‐7.09 (m, 1H, Ar‐H), 5.95 (dd, J = 12.5, 5.2 Hz, 1H, H‐1′), 5.81 (dd, J = 8.1, 2.7 Hz, 1H, H‐5), 5.13‐5.06 (m, 2H, OCH_2_Ph), 4.35‐4.06 (m, 6H, H‐2′, H‐3′, H‐4′, H‐5′, CHCH_3_), 2.85 (q, J = 7.3 Hz, 12H, CH_3_CH_2_NH^+^), 1.45 (dd, J = 7.1, 1.0 Hz, 1.5H, CHCH_3_), 1.34 (dd, J = 7.1, 0.8 Hz, 1H, CHCH_3_), 1.16 (t, J = 7.3 Hz, 18H, CH_3_CH_2_NH^+^). ^31^P NMR (202 MHz, CD_3_OD) δ –7.48 (d, J = 18.4 Hz, 0.5P), –8.13 (d, J = 19.2 Hz, 0.5P), –11.87 (d, J = 21.0 Hz, 0.5P), –11.89 (d, J = 20.3 Hz, 0.5P), –24.32‐(‐24.73) (m, 1P). ^13^C NMR (126 MHz, CD_3_OD) δ 174.8 (d, J_CP_ = 6.1 Hz, C = O), 174.7 (d, J_CP_ = 7.24 Hz, C = O), 166.22 (C = O), 166.19 (C = O), 152.7 (C = O), 152.6 (C = O), 152.41 (d, J_CP_ = 7.2 Hz, C‐Ar ipso OP), 152.36 (d, J_CP_ = 7.1 Hz, C‐Ar ipso OP), 142.8 (C‐6), 137.43 (C‐Ar), 137.41 (C‐Ar), 130.6 (CH‐Ar), 130.52 (CH‐Ar), 130.51 (CH‐Ar), 129.53 (CH‐Ar), 129.52 (CH‐Ar), 129.21 (CH‐Ar), 129.19 (CH‐Ar), 129.18 (CH‐Ar), 129.16 (CH‐Ar), 125.85 (CH‐Ar), 125.84 (CH‐Ar), 125.78 (CH‐Ar), 125.77 (CH‐Ar), 122.0 (d, J_CP_ = 4.8 Hz, CH‐Ar), 121.9 (d, J_CP_ = 5.0 Hz, CH‐Ar), 103.3 (C‐5), 103.2 (C‐5), 89.7 (C‐1′), 89.6 (C‐1′), 85.10 (d, J_CP_ = 7.9 Hz, C‐4′), 85.03 (d, J_CP_ = 7.9 Hz, C‐4′), 75.7 (C‐2′), 75.6 (C‐2′), 71.4 (C‐3′), 71.2 (C‐3′), 67.74 (OCH_2_Ph), 67.68 (OCH_2_Ph), 66.35 (d, J_CP_ = 5.7 Hz, C‐5′), 66.3 (d, J_CP_ = 5.6 Hz, C‐5′), 51.9 (d, J_CP_ = 2.5 Hz, CHCH_3_), 51.6 (d, J_CP_ = 1.9 Hz, CHCH_3_), 47.2 (CH_3_CH_2_NH^+^), 20.80 (d, J_CP_ = 5.3 Hz, CHCH_3_), 20.76 (d, J_CP_ = 6.3 Hz, CHCH_3_), 10.2 (CH_3_CH_2_NH^+^). HRMS‐ESI (m/z): calcd. for C_25_H_31_N_3_O_16_P_3_ [M‐(Et_3_N)_3_+H]^+^ 722.0917, found 722.0917. HPLC reverse‐phase eluting with 0.1 M TEAB buffer, pH 7.4/CH_3_CN from 10/90 to 100/0 in 30 min, F = 1 ml/min, λ = 270 nm, t_R_ 11.534 min (fast‐eluting isomer) and 11.62 min (slow‐eluting isomer), purity 96%.
  Benzyl ((S)‐(((((((2R,3R,4S,5R)‐5‐(6‐amino‐2‐chloro‐9H‐purin‐9‐yl)‐4‐fluoro‐3‐hydroxytetrahydrofuran‐2‐yl)methoxy)(hydroxy)phosphoryl)oxy)(hydroxy)phosphoryl)oxy)(phenoxy)phosphoryl)‐l‐alaninate di(triethylammonium) salt (**7a**). Yield of light yellow oil 23% over 2 steps. ^1^H NMR (500 MHz, CD_3_OD) δ 8.22 (s, 0.5H, H‐8), 8.21 (s, 0.5H, H‐8), 7.22‐7.16 (m, 9H, H‐Ar),7.04‐6.99 (m,1H, H‐Ar) 6.40 (dd, J = 4.2 Hz, J_HF_ = 15.5 Hz, 0.5H, H‐1′), 6.40 (dd, J = 4.3 Hz, J_HF_ = 15.3 Hz, 0.5H, H‐1′), 5.10‐5.08 (m, 0.5H, H‐2′) 5.00‐4.97 (m, 2.5H, H‐2′ and OCH_2_Ph), 4.52 (ddd, J = 3.5, 4.8 Hz, J_HF_ = 20.0 Hz, 1H, H‐3′), 4.26‐4.26 (m, 2H, H‐5′), 4.08‐4.02 (m, 2H, H‐4′, CHCH_3_), 3.02 (q, J = 7.3 Hz, 12H, CH_3_CH_2_NH^+^), 1.35 (d, J = 7.1 Hz, 1.5H, CHCH_3_), 1.24 (d, J = 7.1 Hz, 1H, CHCH_3_), 1.17 (t, J = 7.3 Hz, 18H, CH_3_CH_2_NH^+^). ^31^P NMR (202 MHz, CD_3_OD) δ –7.55 (d, J = 18.3 Hz, 0.5P), –8.15 (d, J = 18.9 Hz, 0.5P), –11.65 (d, J = 20.2, 5.4 Hz, 1P), –24.47 (d, J = 20.2 Hz, 1P). ^19^F NMR (471 MHz, CD_3_OD) δ –199.75 (0.5F), –199.78 (0.5F). ^13^C NMR (126 MHz, CD_3_OD) δ 173.53 (d, J_CP_ = 6.3 Hz, C = O), 173.41 (d, J_CP_ = 7.1 Hz, C = O), 156.68 (C‐6), 154.10 (C‐2), 150.99 (d, J_CP_ = 7.0 Hz, C‐Ar ipso OP), 150.94 (d, J_CP_ = 6.9 Hz, C‐Ar ipso OP), 150.24 (C‐4), 140.58 (CH‐8) 135.99 (C‐Ar), 129.16 (CH‐Ar), 129.11 (CH‐Ar), 128.12 (CH‐Ar), 127.77 (CH‐Ar), 124.46 (CH‐Ar), 124.38 (CH‐Ar), 120.55 (d, J_CP_ = 4.9 Hz, CH‐Ar), 120.53 (d, J_CP_ = 5.0 Hz, CH‐Ar), 117.11 (C‐5), 94.9 (d, J_CF_ = 316 Hz, C‐2′), 82.66 (d, J_CP_ = 5.0 Hz, C‐4′), 82.62 (d, J_CP_ = 5.0 Hz, C‐4′), 82.50 (d, J_CF_ = 16.3 Hz, C‐1′), 74.7 (d, J_CF_ = 24.4 Hz, C‐3′), 74.5 (d, J_CF_ = 24.3 Hz, C‐3′), 66.36 (OCH_2_Ph), 66.31 (OCH_2_Ph), 64.4 (d, J_CP_ = 4.5 Hz, C‐5′), 50.5 (d, J_CP_ = 2.6 Hz, CHCH_3_), 50.21 (d, J_CP_ = 1.8 Hz, CHCH_3_), 47.5 (CH_3_CH_2_NH^+^), 20.79 (d, J_CP_ = 5.5 Hz, CHCH_3_), 20.77 (d, J_CP_ = 6.4 Hz, CHCH_3_), 7.8 (CH_3_CH_2_NH^+^). HPLC reverse‐phase eluting with 0.1 M TEAB buffer, pH 7.4/CH_3_CN from 10/90 to 100/0 in 30 min, F = 1 ml/min, λ = 264 nm, t_R_ 14.17 min, purity 92%. HRMS‐ESI (m/z): calcd. for C_26_H_30_N_6_O_13_FP_3_Cl [M‐(Et_3_N)_3_+H]^+^ 781.0756, found 781.0776.
  Benzyl‐((((((((2R,3S,4R,5R)‐5‐(6‐amino‐9H‐purin‐9‐yl)‐3,4 dihydroxytetrahydrofuran‐2‐yl)methoxy)(hydroxy)phosphoryl)oxy)(hydroxy)phosphoryl)oxy)(phenoxy)phosphoryl)‐l‐alaninate diammonium salt (**8a**). Yield of glassy oil 94%. ^1^H NMR (500 MHz, CD_3_OD) δ 8.54 (d, J = 1.8 Hz, 1H, H‐8), 8.19 (d, J = 1.5 Hz, 1H, H‐2), 7.35‐7.20 (m, 9H, Ar‐H), 7.13‐7.06 (m, 1H, Ar‐H), 6.08 (dd, J = 5.8, 4.6 Hz, 1H, H‐1′), 5.10‐5.06 (m, 2H, OCH_2_Ph), 4.69 (dd, J = 5.7, 5.3 Hz, 0.5H, H‐2′), 4.64 (dd, J = 5.4, 5.4 Hz, 0.5H, H‐2′), 4.51‐4.45 (m, 1H, H‐3′), 4.35‐4.19 (m, 3H, H‐4′, H‐5′), 4.21‐4.08 (m, 1H, CHCH_3_), 1.45 (dd, J = 7.2, 0.9 Hz, 1.5H, CHCH_3_), 1.33 (dd, J = 7.1, 0.9 Hz, 1.5H, CHCH_3_). ^31^P NMR (202 MHz, CD_3_OD) δ –7.52 (d, J = 18.2 Hz, 0.5P), –8.17 (d, J = 19.1 Hz, 0.5P), –11.74 (d, J = 20.7 Hz, 0.5P), –11.77 (d, J = 20.0 Hz, 0.5P), –23.81‐(‐25.02) (m, 1P). ^13^C NMR (126 MHz, CD_3_OD) δ 175.0 (d, J_CP_ = 5.5 Hz, C = O), 174.9 (d, J_CP_ = 6.0 Hz, C = O), 156.84 (C‐2), 156.82 (C‐2), 153.22 (C‐6), 153.18 (C‐6), 152.25 (d, J_CP_ = 7.1 Hz, C‐Ar ipso OP), 152.18 (d, J_CP_ = 7.1 Hz, C‐Ar ipso OP), 150.69 (C‐4), 150.62 (C‐4), 141.3 (C‐8), 137.31 (C‐Ar), 137.28 (C‐Ar), 130.52 (CH‐Ar), 130.49 (CH‐Ar), 129.51 (CH‐Ar), 129.50 (CH‐Ar), 129.19 (CH‐Ar), 129.18 (CH‐Ar), 125.84 (CH‐Ar), 125.84 (CH‐Ar), 125.80 (CH‐Ar), 125.79 (CH‐Ar), 121.84 (d, J_CP_ = 4.9 Hz, CH‐Ar), 121.75 (d, J_CP_ = 4.9 Hz, CH‐Ar), 120.08 (C‐5), 120.06 (C‐5), 89.2 (C‐1′), 89.1 (C‐1′), 85.5 (d, J_CP_ = 9.6 Hz, C‐4′), 85.4 (d, J_CP_ = 9.5 Hz, C‐4′), 76.41 (C‐2′), 76.39 (C‐2′), 71.9 (C‐3′), 71.7 (C‐3′), 67.84 (OCH_2_Ph), 67.78 (OCH_2_Ph), 66.5 (d, J_CP_ = 5.9 Hz, C‐5′), 66.4 (d, J_CP_ = 6.0 Hz, C‐5′), 51.8 (d, J_CP_ = 2.5 Hz, CHCH_3_), 51.6 (d, J_CP_ = 1.5 Hz, CHCH_3_), 20.74 (d, J_CP_ = 4.8 Hz, CHCH_3_), 20.69 (d, J_CP_ = 4.4 Hz, CHCH_3_). HRMS‐ESI (m/z): calcd. for C_26_H_32_N_6_O_14_P_3_ [M‐NH_3_)_2_+H]^+^ 745.1189, found 745.1197.

## PREPARATION OF TRIPHOSPHATE ARYLOXY PHOSPHORAMIDATE OF ADENOSINE WITH CONVENTIONAL HEATING

**Figure 2:**
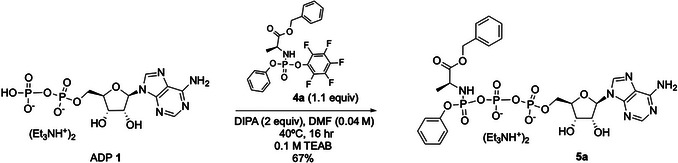
Synthesis of adenosine γ‐ProTriP (**5a**) with conventional heating.

This alternate protocol describes the synthesis of adenosine γ‐ProTriP (**5a**), obtained in a 67% yield, through the reaction of the pentafluorophenyl phosphorylating reagent **4a** with adenosine diphosphate (ADP, **1**) in the presence of diisopropylamine (DIPA) in DMF at 40°C under conventional heating for 16 hr (Fig. 2; Tisnerat et al., 2025).

### Additional Materials (also see Basic Protocol)

Magnetic stirrer heating plate with temperature probe
Heating block for magnetic stirrer

1 Suspend 50 mg (1 equiv, 0.12 mmol) ADP (**1**) and 65 mg (1.1 equiv, 0.13 mmol) **4a** in anhydrous DMF (3 ml) under a nitrogen atmosphere in a 10‐ml one‐neck round‐bottom flask containing a magnetic stirrer.
2 Add 34 µl (2 equiv, 0.24 mmol) DIPA.
  A cloudy suspension forms.
3 Stir and heat the reaction for 16 hr at 40°C.
  A clear solution is obtained.
4 Evaporate the solvent using a rotary evaporator under reduced pressure with a nitrogen trap.
5 Dissolve the crude product in 0.1 M TEAB buffer (1 ml) and sonicate the resulting suspension to reduce particle size. If needed, scrape the precipitate with a spatula and repeat sonication.
  Pentafluorophenol precipitates.
6 Carefully filter this suspension through a 0.2‐µm PTFE filter using a 2‐ml syringe.
7 Purify the crude product by automated flash chromatography with a Biotage Isolera One chromatographic system fitted with a Biotage SNAP KP‐C18‐HS (60 g) column, using a solvent gradient from 95:5 (v/v) to 0:100 (v/v) of 0.1 M TEAB buffer, pH 7.4/acetonitrile over 40 min at 50 ml/min flow rate. Monitor at two absorbance wavelengths, λ = 254 nm and 263 nm.
8 Combine the fractions containing pure product and evaporate to dryness using a rotary evaporator under reduced pressure to obtain **5a** as clear oil.
9 Dry the pure product under vacuum overnight to obtain **5a** as a solid.
10 Characterize the compound by ^1^H, ^13^C, and ^31^P NMR, HRMS, and HPLC.
  Benzyl‐((((((((2R,3S,4R,5R)‐5‐(6‐amino‐9H‐purin‐9‐yl)‐3,4 dihydroxytetrahydrofuran‐2‐yl)methoxy)(hydroxy)phosphoryl)oxy)(hydroxy)phosphoryl)oxy)(phenoxy)phosphoryl)‐l‐alaninate di(triethylammonium) salt (**5a**). Yield of white solid 67%. ^1^H NMR (500 MHz, CD_3_OD) δ 8.54 (d, J = 1.8 Hz, 1H, H‐8), 8.19 (d, J = 1.5 Hz, 1H, H‐2), 7.35‐7.20 (m, 9H, Ar‐H), 7.13‐7.06 (m, 1H, Ar‐H), 6.08 (dd, J = 5.8, 4.6 Hz, 1H, H‐1′), 5.10‐5.06 (m, 2H, OCH_2_Ph), 4.69 (dd, J = 5.7, 5.3 Hz, 0.5H, H‐2′), 4.64 (dd, J = 5.4, 5.4 Hz, 0.5H, H‐2′), 4.51‐4.45 (m, 1H, H‐3′), 4.35‐4.19 (m, 3H, H‐4′, H‐5′), 4.21‐4.08 (m, 1H, CHCH_3_), 2.97 (q, J = 7.3 Hz, 12H, CH_3_CH_2_NH^+^), 1.45 (dd, J = 7.2, 0.9 Hz, 1.5H, CHCH_3_), 1.33 (dd, J = 7.1, 0.9 Hz, 1.5H, CHCH_3_), 1.20 (t, J = 7.3 Hz, 18H, CH_3_CH_2_NH^+^). ^31^P NMR (202 MHz, CD_3_OD) δ –7.52 (d, J = 18.2 Hz, 0.5P), –8.17 (d, J = 19.1 Hz, 0.5P), –11.74 (d, J = 20.7 Hz, 0.5P), –11.77 (d, J = 20.0 Hz, 0.5P), –23.81‐(‐25.02) (m, 1P). ^13^C NMR (126 MHz, CD_3_OD) δ 174.9 (d, J_CP_ = 6.1 Hz, C = O), 174.8 (d, J_CP_ = 7.1 Hz, C = O), 157.3 (C‐2), 153.82 (C‐6), 153.80 (C‐6), 152.4 (d, J_CP_ = 6.8 Hz, C‐Ar ipso OP), 152.3 (d, J_CP_ = 6.4 Hz, C‐Ar ipso OP), 151.0 (C‐4), 150.9 (C‐4), 141.13 (C‐8), 141.10 (C‐8), 137.40 (C‐Ar), 137.38 (C‐Ar), 130.52 (CH‐Ar), 130.52 (CH‐Ar), 130.49 (CH‐Ar), 130.48 (CH‐Ar), 129.5 (CH‐Ar), 129.2 (CH‐Ar), 125.81 (CH‐Ar), 125.79 (CH‐Ar), 125.74 (CH‐Ar), 125.73 (CH‐Ar), 121.95 (d, J_CP_ = 4.8 Hz, CH‐Ar), 121.91 (d, J_CP_ = 4.8 Hz, CH‐Ar), 120.17 (C‐5), 120.14 (C‐5), 88.9 (C‐1′), 88.8 (C‐1′), 85.7 (d, J_CP_ = 8.8 Hz, C‐4′), 85.6 (d, J_CP_ = 8.7 Hz, C‐4′), 76.13 (C‐2′), 76.09 (C‐2′), 72.16 (C‐3′), 71.96 (C‐3′), 67.73 (OCH_2_Ph), 67.69 (OCH_2_Ph), 66.8 (d, J_CP_ = 5.9 Hz, C‐5′), 66.7 (d, J_CP_ = 6.0 Hz, C‐5′), 51.9 (d, J_CP_ = 2.7 Hz, CHCH_3_), 51.6 (d, J_CP_ = 1.5 Hz, CHCH_3_), 47.31 (CH_3_CH_2_NH^+^), 20.81 (CHCH_3_), 20.76 (CHCH_3_), 9.74 (CH_3_CH_2_NH^+^). HRMS‐ESI (m/z): calcd. for C_26_H_32_N_6_O_14_P_3_ [M‐(Et_3_N)_2_+H]^+^ 745.1190, found 745.1189. HPLC reverse‐phase eluting with 0.1 M TEAB buffer, pH 7.4/CH_3_CN from 10/90 to 100/0 in 30 min, F = 1 ml/min, λ = 263 nm, t_R_ 8.507 min, purity 95%.

## Support Protocol 1 CATION EXCHANGE OF UDP DISODIUM SALT TO UDP DI(TRIETHYLAMONIUM) SALT

**Figure 3:**
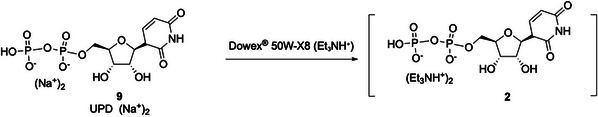
Displacement of the disodium salt of UDP (**9**) on cation‐exchange resin (Dowex 50W‐X8) to obtain UDP di(triethylammonium) salt (**2**).

Under the conditions described in Basic Protocol, no reaction was observed when using the disodium salt of adenosine diphosphate (ADP) or uridine diphosphate (UDP). This lack of reactivity is most likely due to the poor solubility of these nucleotide analogues in DMF. Because the acid form of UDP is not commercially available, Support Protocol 1 outlines a method for converting UDP disodium salt (**9**) into its di(triethylammonium) form (**2**; Fig. 3). The conversion is achieved through ion exchange using a cation exchange resin preconditioned with triethylammonium ions.

### Materials

Dowex 50W‐X8 (H^+^) resin (Sigma‐Aldrich)
Methanol (MeOH; Sigma‐Aldrich)
Deionized water
Triethylamine (Et_3_N; Sigma‐Aldrich)
Uridine diphosphate (UDP) disodium salt **9** (Merck, cat. no. FLUH9AD3CA06)
Nitrogen
Schlenk line with high vacuum pump and nitrogen trap
Liquid nitrogen

10‐mm‐diameter glass column
10‐ml glass tubes
100‐ml one‐neck round‐bottom flasks
Rotary evaporator equipped with vacuum pump and nitrogen trap
Analytical thin‐layer chromatography plates: aluminum‐backed TLC plates, precoated with silica gel 60 F254, 0.2 mm (Merck Kieselgel)
UV light source
Nuclear magnetic resonance (NMR) instrument

1 Add Dowex 50W‐X8 (H^+^) resin to a 10‐mm‐diameter glass column to obtain a bed of 55 mm.
2 Wash the resin with methanol followed by deionized water (3 bed volumes each).
3 Equilibrate the resin with a solution of 60% Et_3_N in water (3 bed volumes).
4 Wash with deionized water until pH is 7.0 (9 bed volumes).
  The eluent color will change from light red to colorless.
5 Solubilize 100 mg UDP disodium salt **9** in 5 ml deionized water.
6 Load the uridine diphosphate sodium salt solution on the top of the column and drain into 10‐ml glass tubes.
7 Rinse the column with 5 ml deionized water and collect (3 bed volumes).
8 Spot all collected fractions on a TLC plate and combine the tubes containing the product (identified by their absorbance under UV light) into a 100‐ml one‐neck round‐bottom flask.
9 Concentrate the solution using a rotary evaporator under reduced pressure with a nitrogen trap until only a few drops remain.
10 Freeze in liquid nitrogen and dry under vacuum overnight to obtain the di(triethylammonium) salt of UDP **2** as a solid.
11 Characterize the compound by ^1^H and ^31^P NMR.
  This compound is used directly, without further purification, in the Basic Protocol.
  Uridine diphosphate di(triethylammonium) salt (**2**). ^1^H NMR (500 MHz, CD_3_OD) δ 8.10‐8.02 (m, 1H, H‐6), 5.98‐5.92 (m, 1H, H‐1′), 5.85‐5.79 (m, 1H, H‐5), 4.38‐4.31 (m, 1H, H‐2′), 4.30‐4.17 (m, 3H, H‐4′, H‐5′), 4.14‐4.08 (m, 1H, H‐3′), 3.15 (q, J = 7.2 Hz, 12H, CH_3_CH_2_NH^+^), 1.30 (t, J = 6.6 Hz, 18H, CH_3_CH_2_NH^+^). ^31^P NMR (202 MHz, CD_3_OD) δ –9.95 (d, J = 20.4 Hz, 1P), –11.14 (d, J = 20.4 Hz, 1P).

## Support Protocol 2 SYNTHESIS OF DI(TRIETHYLAMMONIUM) SALT OF CLOFARABINE 5′‐DIPHOSPHATE (3)

**Figure 4:**
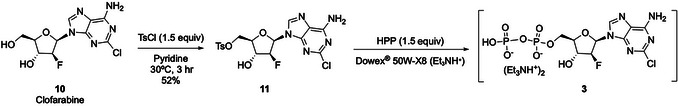
Synthesis of clofarabine 5′‐diphosphate di(triethylammonium) salt (**3**).

This protocol describes the synthesis of the di(triethylammonium) salt of clofarabine 5′‐diphosphate (**3**; Fig. 4). Briefly, the primary alcohol of clofarabine (**10**) is first activated with tosyl chloride, affording the intermediate **11** (Blackburn & Langston, 1991). Subsequent phosphorylation of **11** with tris(tetrabutylammonium) hydrogen pyrophosphate (HPP) affords clofarabine 5′‐diphosphate (**3**) as the di(triethylammonium) salt after exchange of the tetrabutylammonium salt on a cation‐exchange resin in the triethylammonium form (Davisson et al., 1987). Compound **3** is used without further purification to obtain the corresponding γ‐ProTriP (**7a**) following the Basic Protocol.

### Materials

Clofarabine **10** (Carbosynth Limited)
Anhydrous pyridine (Sigma‐Aldrich)
Deionized water
Dichloromethane (DCM; Sigma‐Aldrich)
Saturated aqueous bicarbonate solution
Magnesium sulfate (MgSO_4_; Sigma‐Aldrich)
Methanol (MeOH; Sigma‐Aldrich)
Celite
Acetonitrile, HPLC grade (Sigma‐Aldrich)
Tris(tetrabutylammonium) hydrogen pyrophosphate (HPP; Sigma‐Aldrich)
Dowex 50W‐X8 (H^+^) resin (Sigma‐Aldrich)
Triethylamine (Et_3_N; Sigma‐Aldrich)
Tosyl chloride (TsCl; Sigma‐Aldrich)

Nitrogen
5‐, 25‐, and 100‐ml one‐neck round‐bottom flasks
Magnetic stirrer
Schlenk line with vacuum pump and nitrogen trap
Stirring heating plate with temperature probe
Ice
250‐ml separating funnel
Rotary evaporator equipped with vacuum pump and nitrogen trap
Funnel
Cotton
Reverse‐phase chromatography column: Biotage SNAP KP‐C18‐HS 60 g cartridge
Flash chromatography apparatus: Biotage Isolera One
UV light source
Nuclear magnetic resonance instrument
Mass spectrometry instrument
10‐mm‐diameter glass column
10‐ml glass tubes

Liquid nitrogen
Analytical thin‐layer chromatography plates: aluminum‐backed TLC plates, precoated with silica gel 60 F254, 0.2 mm (Merck Kieselgel)

#### Prepare clofarabine 5′‐tosylate (11)

1 Solubilize 200 mg (1 equiv, 0.66 mmol) clofarabine (**10**) in 4 ml pyridine under a nitrogen atmosphere in a 25‐ml one‐neck round‐bottom flask containing a magnetic stirrer.
2 Cool the solution to 0°C using an ice bath.
3 Add 189 mg (1.5 eq, 0.99 mmol) tosyl chloride.
4 Stir for 10 min at 0°C, and then heat the solution at 30°C for 3 hr.
  The reaction mixture turns yellow.
5 Cool the solution to 0°C with an ice bath and add a few pieces of ice and 40 ml deionized water.
6 Transfer the mixture to a 250‐ml separating funnel and extract the product four times with 40 ml DCM each time.
7 Combine all organic phases and wash with 40 ml saturated aqueous sodium bicarbonate solution.
8 Dry the organic phase over MgSO_4_, filter through cotton, and evaporate to dryness using a rotary evaporator.
9 Solubilize the crude product in DCM with a minimum amount of MeOH.
10 Add Celite and evaporate using a rotary evaporator to obtain a dry load.
11 Purify the crude product by automated flash chromatography with a Biotage Isolera One chromatographic system fitted with a Biotage Sfär silica (25 g) column, using a gradient from 100:0 (v/v) to 90:10 (v/v) DCM/MeOH over 25 min at 80 ml/min flow rate. Monitor at two absorbance wavelengths, λ = 254 nm and 280 nm.
12 Combine the fractions containing the pure product, evaporate to dryness using a rotary evaporator under reduced pressure, and dry under high vacuum overnight to obtain **11** as solid.
13 Characterize the compound by ^1^H, ^13^C, and ^19^F NMR and HRMS.
  5‐(6‐Amino‐2‐chloro‐9H‐purin‐9‐yl)‐4‐fluoro‐3‐hydroxytetrahydrofuran‐2‐yl)methyl 4‐methyl benzenesulfonate (**10**). Yield of white solid 52%. ^1^H NMR (400 MHz, acetone‐d_6_) δ 7.96 (d, J = 2.3 Hz, 1H, H‐8), 7.83‐7.75 (m, 2H, Ar‐H), 7.44‐7.34 (m, 2H, Ar‐H), 7.07 (bs, 2H, NH_2_), 6.40 (dd, J = 15.7, 4.3 Hz, 1H, H‐1′), 5.36 (d, J = 4.9 Hz, 1H, OH), 5.34 (dd, J = 4.3, 3.4 Hz, 0.5H, H‐2′), 5.21 (dd, J = 4.2, 3.5 Hz, 0.5H, H‐2′), 4.75‐4.63 (m, 1H, H‐3′), 4.41 (d, J = 4.8 Hz, 1H, H‐5′), 4.25‐4.16 (m, 1H, H‐4′), 2.41 (s, 3H, CH_3_). ^13^C NMR (101 MHz, acetone‐d6) δ 157.9 (C‐6), 154.8 (C‐2), 151.5 (C‐4), 146.1 (C‐Ar), 141.1 (d, J = 4.8 Hz, C‐8), 133.8 (C‐Ar), 130.7 (CH‐Ar), 128.8 (CH‐Ar), 118.9 (C‐5), 95.9 (d, J = 192.5 Hz, C‐2′), 83.5 (d, J = 16.8 Hz, C‐1′), 81.7 (d, J = 4.9 Hz, C‐4′), 74.9 (d, J = 24.7 Hz, C‐3′), 70.1 (d, J = 2.2 Hz, C‐5′), 21.5 (CH_3_). ^19^F NMR (376 MHz, acetone‐d6) δ –199.40. HRMS‐ESI (m/z): calcd. for C_17_H_18_N_5_O_5_SClF [M+H]^+^ 458.0701, found 458.0701.

#### Prepare clofarabine 5′‐diphosphate di(triethylammonium) salt (3)

14 Suspend 50 mg (1 eq, 0.11 mmol) **11** in acetonitrile under a nitrogen atmosphere in a 5‐ml one‐neck round‐bottom flask.
15 Add 200 mg (2 eq, 0.22 mmol) tris(tetrabutylammonium) hydrogen pyrophosphate (HPP).
16 Stir at room temperature for 3 days.
  The reaction mixture will turn green and then yellow.
17 Evaporate to dryness using a rotary evaporator.
18 Solubilize the crude product in 1 ml deionized water in a 5‐ml one neck round‐bottom flask.
19 Add Dowex 50W‐X8 (H^+^) resin to a 10‐mm‐diameter glass column to obtain a bed of 80 mm.
20 Wash the resin with 3 bed volumes of methanol followed by 3 bed volumes of deionized water.
21 Equilibrate the resin with 3 bed volumes of 60% Et_3_N in water.
22 Wash with deionized water until pH is 7.0 (9 bed volumes).
  The eluent color will change from light red to colorless.
23 Load the solution of crude product onto the top of the column and drain into 10‐ml glass tubes.
24 Rinse the column with 5 ml deionized water (3 bed volumes) and collect.
25 Spot all collected fractions on a TLC plate and combine the tubes containing the product (identified by their absorbance under UV light) into a 100‐ml one‐neck round‐bottom flask.
26 Concentrate the solution using a rotary evaporator under reduced pressure with a nitrogen trap until only a few drops remain.
27 Freeze in liquid nitrogen and dry under vacuum overnight to obtain di(triethylammonium) salt **3** as solid.
28 Characterize the compound by ^31^P NMR.
  The compound is used without any further purification in the Basic Protocol.
  (((2R,3S,4R,5R)‐5‐(6‐Amino‐2‐chloro‐9H‐purin‐9‐yl)‐4‐fluoro‐3‐hydroxytetrahydrofuran‐2‐yl)methyl diphosphate) di(triethylammonium) salt (**3**). ^31^P NMR (202 MHz, CD_3_OD) δ –11.11 (d, J = 18.3 Hz, 1P), –12.19 (d, J = 18.3 Hz, 1P).

## Support Protocol 3 SYNTHESIS OF PENTAFLUOROPHENYL PHOSPHORYLATING REAGENTS

**Figure 5:**
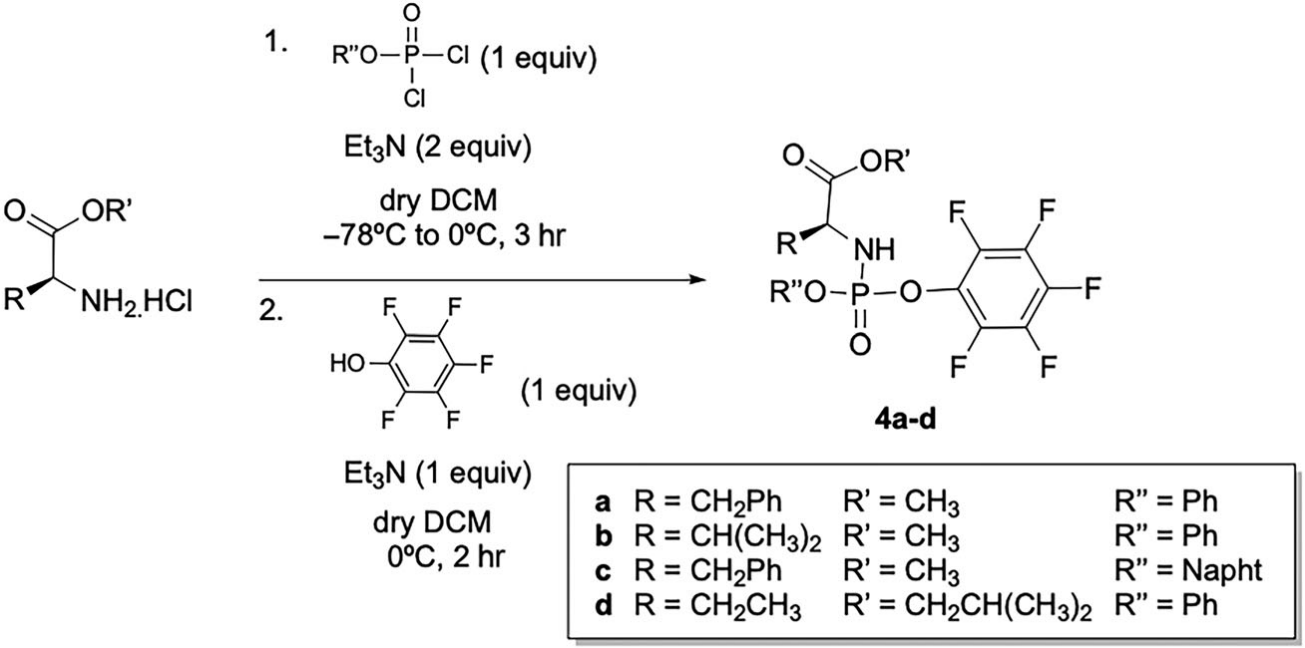
Synthesis of pentafluorophosphorylation reagents **4a**‐**d**.

This protocol outlines the synthesis of amino ester pentafluorophenyl phosphorylating reagents **4a**‐**d** (Fig. 5), which serve as pivotal intermediates in the construction of triphosphate aryloxy phosphoramidate prodrugs (γ‐ProTriPs). The amino acid moiety is derived from naturally occurring α‐amino acids, with l‐alanine being the preferred scaffold, as it is consistently employed in all clinically advanced aryloxy phosphoramidate therapeutics. A selection of short‐chain esters, including linear (ethyl), branched (isopropyl), and benzylic variants, are utilized, while phenol and 1‐naphthol serve as the aryl components.

### Materials

l‐Alanine benzyl ester hydrochloride (Sigma‐Aldrich, cat. no. ENAH93E74A3E), l‐alanine isopropyl ester hydrochloride (BioSynth, cat. no. MFCD08059709), *or* l‐leucine ethyl ester hydrochloride (Sigma‐Aldrich, cat. no. S598895)
Anhydrous dichloromethane (DCM; Sigma‐Aldrich)
–78°C dry ice/acetone and 0°C ice/water cooling baths
Anhydrous triethylamine (Sigma‐Aldrich)
Phenyl dichlorophosphate (Sigma‐Aldrich, cat. no. P22389)
1‐Naphthyl dichlorophosphate (prepared as described in Serpi et al., 2013)
Pentafluorophenol (Sigma‐Aldrich, cat. no. 103799)
Anhydrous diethyl ether (Et_3_N; Fisher)
Hexanes (Fisher)
Ethyl acetate (Fisher)
Celite

50‐, 100‐, and 250‐ml one‐neck round‐bottom flasks
Nitrogen
Magnetic stirrer
Stirring plate
Dewar
1‐, 2‐, and 10‐ml syringes
Nuclear magnetic resonance (NMR) instrument
250‐ml conical vacuum flask
Glass‐sintered filter funnel
Rotary evaporator equipped with a vacuum pump
Normal phase column chromatography apparatus: Biotage Sfär 50‐g cartridge
Flash chromatography apparatus: Biotage Isolera One
Analytical TLC plates: aluminum‐backed TLC plates, precoated with silica gel 60 F254, 0.2 mm (Merck Kieselgel)
UV light source

1 Solubilize under a nitrogen atmosphere in a 50‐ml one‐neck round‐bottom flask containing a magnetic stirrer on a magnetic plate:
  a. For **4a** or **4d**: 1 g (1 equiv, 4.64 mmol) l‐alanine benzyl ester hydrochloride in anhydrous DCM (6 ml).
  b. For **4b**: 1 g (1 equiv, 5.96 mmol) l‐alanine isopropyl ester hydrochloride in anhydrous DCM (8 ml).
  c. For **4c**: 1 g (1 equiv, 5.11 mmol) l‐leucine ethyl ester hydrochloride in anhydrous DCM (7 ml).
2 Cool the mixture to –78°C using a dry ice/acetone bath in a Dewar.
3 Add dropwise successively:
  a. For **4a**: 1.3 ml (2 equiv, 9.28 mmol) anhydrous Et_3_N with a 2‐ml syringe and 690 µl (1 equiv, 4.64 mmol) phenyl dichlorophosphate with a 1‐ml syringe.
  b. For **4b**: 1.7 ml (2 equiv, 11.92 mmol) anhydrous Et_3_N with a 2‐ml syringe and 890 µl (1 equiv, 5.96 mmol) phenyl dichlorophosphate with a 1‐ml syringe.
  c. For **4c**: 1.4 ml (2 equiv, 10.22 mmol) anhydrous Et_3_N with a 2‐ml syringe and 760 µl (1 equiv, 5.11 mmol) phenyl dichlorophosphate with a 1‐ml syringe.
  d. For **4d**: 1.3 ml (2 equiv, 9.28 mmol) anhydrous Et_3_N with a 2‐ml syringe and 1.22 g (1 equiv, 4.64 mmol) 1‐naphthyl dichlorophosphate.
4 Stir the resulting mixture for 30 min at –78°C, and then allow to warm to 0°C in an ice/water bath over 3 hr.
  A white suspension forms.
5 Once ^31^P‐NMR confirms completion of the reaction (disappearance of the singlet at 3.58 ppm), prepare the following solution, under a nitrogen atmosphere, in a 10‐ml one‐neck round‐bottom flask containing a magnetic stirrer:
  a. For **4a** or **4d**: 854 mg (1 equiv, 4.64 mmol) pentafluorophenol and 710 µl (1.1 equiv, 5.10 mmol) anhydrous Et_3_N in anhydrous DCM (6 ml).
  b. For **4b**: 1.1 g (1 equiv, 5.96 mmol) pentafluorophenol and 710 µl (1.1 equiv, 5.10 mmol) anhydrous Et_3_N in anhydrous DCM (8 ml).
  c. For **4c**: 940 mg (1 equiv, 5.11 mmol) pentafluorophenol and 914 µl (1.1 equiv, 6.56 mmol) anhydrous Et_3_N in anhydrous DCM (7 ml).
6 Stir this solution for a few minutes.
7 Using a 10‐ml syringe, add this solution dropwise over 15 min to the mixture from step 4 under a nitrogen atmosphere, keeping the mixture at 0°C in the ice/water bath.
8 Stir for 2 hr.
9 Filter off the triethylamine hydrochloride using a glass‐sintered filter funnel and a 250‐ml conical vacuum flask and wash with ∼20 ml DCM followed by 20 ml diethyl ether.
10 Evaporate the filtrate under reduced pressure on a rotary evaporator.
11 Purify the crude product by flash chromatography using a Biotage Sfär silica (50 g) column with a gradient of:
  a. For **4a**: ethyl acetate in hexanes from 0:100 (v/v) to 30:70 (v/v), flow 50 ml/min in 12 column volumes (CV; 1 CV = 30 ml).
  b. For **4b**: ethyl acetate in hexanes from 20:80 (v/v) to 40:60 (v/v) flow 50 ml/min in 12 CV.
  c. For **4c**: ethyl acetate in DCM from 0:100 (v/v) to 10:100 (v/v), flow 50 ml/min in 12 CV.
  d. For **4d**: DCM in hexanes from 0:100 (v/v) to 100:0 (v/v), flow 50 ml/min in 12 CV.
12 Monitor the fractions by TLC:
  a. For **4a**, **4b**, or **4d**: using 8:2 (v/v) hexanes/ethyl acetate as the elution solvent and visualizing under UV light (*R*_f_ = 0.4).
  b. For **4c**: using 95:5 (v/v) DCM/ethyl acetate as the elution solvent and visualizing under UV light (*R*_f_ = 0.8).
13 Combine the fractions containing pure product and evaporate to dryness under reduced pressure on a rotary evaporator to obtain the pentafluorophenyl phosphorylating reagents **4a**, **4b**, or **4c** as white solids or **4d** as a brown oil, in each case as a 1:1 mixture of diastereoisomers.
14 Characterize the compounds by ^1^H and ^31^P NMR.
  The compounds are used in the Basic Protocol and/or Alternate Protocol.
  (S)‐2‐[‐(S)‐(2,3,4,5,6‐Pentafluoro‐phenoxy)‐phenoxy‐phosphorylamino]propionic acid benzyl ester (**4a**). Yield of a white solid 64%. ^1^H NMR (400 MHz, CDCl_3_) δ 7.51‐6.95 (m, 10H, H‐Ar), 5.09 (d, J = 10.2 Hz, 2H, CH_2_Ph), 4.23‐4.03 (m, 1H, CHCH_3_), 1.31 (d, J = 63.2 Hz, 3H, CHCH_3_). ^31^P NMR (162 MHz, CDCl_3_) δ –1.93‐(–2.01).
  (S)‐2‐[‐(S)‐(2,3,4,5,6‐Pentafluoro‐phenoxy)‐phenoxy‐phosphorylamino]propionic acid isopropyl ester (**4b**). Yield of a white solid 37%.; ^1^H NMR (CDCl_3_, 400 MHz) δ 7.38‐7.34 (m, 2 H, Ar‐H), 7.27‐7.24 (m, 2 H, Ar‐H), 7.23‐7.19 (m, 1 H, Ar‐H), 5.04 (m, 1 H, ‐CH(CH_3_)_2_), 4.18‐4.09 (m, 0.5 H, CHCH_3_), 4.00‐3.95 (m, 0.5 H, CHCH_3_), 1.45‐1.24 (m, 9H, CHCH_3_, ‐CH(CH_3_)_2_); ^31^P NMR (CDCl_3_, 162 MHz) δ −0.50‐(–0.56).
  Ethyl ((perfluorophenoxy)(phenoxy)phosphoryl)‐l‐leucinate (**4c**). Yield of a white solid 61%. ^1^H NMR (500 MHz, CDCl_3_) δ 7.39‐7.31 (m, 2H, Ar‐H), 7.30‐7.17 (m, 3H, Ar‐H), 4.24‐4.05 (m, 3H), 3.86‐3.68 (m, NH), 1.77‐1.49 (m, 3H), 1.26 (dt, J = 9.7, 7.1 Hz, 3H CH_2_CH_3_), 0.95‐0.87 (m, 6H, ‐CH_2_CH(CH_3_)_2_). ^31^P (202 MHz, CDCl_3_) δ –1.23‐(–1.50).
  N‐[(S)‐(1‐Naphthalenyloxy)(2,3,4,5,6‐pentafluorophenoxy)phosphinyl]‐l‐alanine phenylmethyl ester (**4d**). Yield of a brown oil 43%. NMR. ^1^H NMR (500 MHz, CDCI_3_): δ 8.13‐8.10 10 (1 H, m, H‐Ar), 7.90‐7.88 (1H, m, Ar‐H), 7.73 (1H, m, Ar‐H), 7.62‐ 7.55 (3H, m, Ar‐H), 7.45‐7.41 (1H, m, Ar‐H), 7.36‐7.28 (5H, m, Ar‐H), 5.05 (2H, m, CH_2_Ph), 4.38‐4.08 (1H, m, CHCH_3_), 1.49 (1.5H, d, J = 3.5 Hz, CHCH_3_), 1.47 (1.5H, d, J = 3.5 Hz, CHCH_3_). ^31^P NMR (202 MHz, CDCI_3_): δ –1.35‐(–1.41).

## REAGENTS AND SOLUTIONS

### TEAB buffer, 0.1 M

10 g (13.9 ml) of 0.1 M triethylamine (Et_3_N; Sigma‐Aldrich)
1 L deionized water
Mix thoroughly
Bubble carbon dioxide gas through the solution until pH reaches 7.4
Store up to 1 month in a screw‐cap bottle at 4°C

## COMMENTARY

### Background Information

To date, more than 30 nucleoside and nucleotide analogues have received regulatory approval for therapeutic use across a broad spectrum of diseases, including viral infections, various forms of cancer, parasitic diseases, and both bacterial and fungal infections (Hruba et al., 2023; Roy & Agrofoglio, 2022; Serpi et al., 2016). In addition to the compounds currently available on the market, numerous analogues are undergoing evaluation in clinical and preclinical trials, underscoring the continued innovation and expansion within this class of therapeutics.

A key challenge to the pharmacological efficacy of NAs is their slow and often inefficient phosphorylation, which is essential for activation (Van Rompay et al., 2000). Recent recognition that many NAs have rate‐limiting second and third phosphorylation steps has prompted the development of highly phosphorylated NA prodrugs (Gollnest et al., 2016; Meier, 2017). Triphosphate nucleoside prodrugs are synthesized via bioreversible modification of the γ‐phosphate group. Specifically, this involves the incorporation of acyloxybenzyl (Gollnest et al., 2015; Jia et al., 2020), alkyl (Jia et al., 2023), or mixed non‐symmetric acyloxybenzyl‐alkyl moieties (Zhao et al., 2020), resulting in TriPPPro compounds, as well as the addition of an amino acid and an aryloxy group, forming γ‐ProTriP derivatives (Tisnerat et al., 2025). These masking groups are cleaved enzymatically or chemically within the cell, releasing the active triphosphate nucleoside intracellularly. TriPPPro compounds were first exemplified through modifications to the anti‐HIV nucleoside stavudine (Zhao et al., 2020), which were followed by applications to 3′‐fluoro‐3′‐deoxythymidine (Weising et al., 2022) and various other nucleoside analogues. These compounds demonstrated high potency in inhibiting HIV replication and were shown, in cell extract studies, to release the active triphosphate species independently of kinase‐mediated phosphorylation. Recent research has further expanded the therapeutic potential of the TriPPPro strategy to the treatment of malaria, representing a promising direction for future anti‐*Plasmodium falciparum* drug development (Nikolova et al., 2025).

γ‐ProTriP derivatives, more recently reported, have been applied to FDA‐approved anticancer NAs such as clofarabine and gemcitabine (Tisnerat et al., 2025). These triphosphate prodrugs were shown to be chemically robust at physiologically relevant pH values, while also moderately stable in rat serum. Clofarabine γ‐ProTriP displayed remarkable *in vitro* anticancer activity against a panel of tumor cell lines.

Collectively, these investigations pave the way for the development of more effective nucleotide‐based therapeutics. The ability to deliver nucleotide triphosphate analogues directly into cells offers a valuable tool for both biochemical research and clinical applications, with the potential to significantly enhance treatment outcomes across multiple disease areas.

### Critical Parameters

The synthetic procedures outlined in this unit should only be performed by individuals with prior training in experimental organic chemistry, who are therefore familiar with common laboratory techniques such as solvent evaporation, extraction, TLC, column chromatography, and spectroscopy, as well as one‐dimensional (^1^H, ^13^C, ^31^P) and two‐dimensional (COSY, HSQC, and HMBC) NMR experiments, along with mass spectrometry.

When hazardous materials are involved, ensuring laboratory safety is of the highest priority; thus, strict adherence to the reported procedures is strongly advised.

The starting nucleosides in their sodium salt forms did not react under the conditions of the Basic Protocol or Alternate Protocol due to poor solubility of the nucleotide.

Exchange of UDP sodium salt to UDP leads to decomposition of the diphosphate to monophosphate

To stabilize the two anhydride bonds, the α and β phosphate groups must remain partially ionized.

The γ‐ProTriPs are moisture‐sensitive compounds; thus, they must be handled under inert atmosphere and stored at –20°C.

### Troubleshooting

Refer to Table 1 to troubleshoot specific aspects of the procedures during phosphorylation.

**Table 1:** Troubleshooting Guide for Phosphorylation

Problem	Possible cause	Solution
Formation of diphosphate prodrugs as side product	The reaction temperature exceeded 40°C, leading to decomposition of the nucleotide diphosphate.	Strictly maintain the reaction temperature at 40°C, and store either commercially available or synthesized nucleoside diphosphates in a freezer at –20°C.
γ‐ProTriP obtained as a mixture of diastereoisomers (*S*_P_/*R*_P_ = 2:1), even when the nucleotide is reacted with a phosphorylating reagent as single diastereoisomer (*S*_P_).	Partial isomerization at the phosphorus center occurred under the conditions of the Basic Protocol or Alternate Protocol.	Follow Support Protocol 1 to prepare the di (triethylammonium) salt of the nucleotide and react this, instead of the acid form, with the pentafluorophenol phosphorylating reagent following either Basic Protocol or Alternate Protocol, to minimize isomerization (*S*_P_/*R*_P_ = 9:1).
Low yields of γ‐ProTriPs from synthesized diphosphate nucleoside analogues	Conversion of the tosylated intermediate into the corresponding diphosphate salt was inefficient.	Purify the nucleoside diphosphate before the reaction with the phosphorylating reagent.

### Understanding Results

This article describes the synthesis of nucleotide prodrugs in which the γ‐phosphate of a nucleotide is masked with an aryloxy moiety and an amino acid ester. The starting 5′‐diphosphate nucleosides can be synthesized from the corresponding 5′‐tosylate analogues using tetrabutylammonium hydrogen pyrophosphate, and the phosphorylating reagent can be synthesized by reacting pentafluorophenol with aryloxy phosphoramidate chloride prepared *in situ*. The 5′‐diphosphate nucleoside and the phosphorylating reagent are then reacted in DMF at 40°C, either under conventional heating overnight or via microwave irradiation for 3 hr.

The formation of the prodrug is monitored by ^31^P NMR, by observing both the disappearance of the two doublet peaks at approximately δ = −10.8 and −11.3 ppm, corresponding to the α and β phosphorus atoms of the nucleoside, respectively, and the appearance of the three characteristic signals at approximately δ = −7.1 (two doublet peaks), −12.5 (two doublet peaks), and −23.8 ppm (a multiplet peak), corresponding to the three phosphorus atoms of the triphosphate prodrug. The product is obtained as a mixture of two diastereoisomers (ratio 1:1) due to the newly formed chiral center at the γ‐phosphorus atom, which explains the twin peaks observed in the phosphorus NMR.

The synthesized triphosphate prodrugs are purified by RP‐HPLC and characterized by ^1^H, ^13^C, ^31^P NMR, and mass spectrometry. The average yield is ∼45%‐80%, and prodrugs typically show >90% purity.

### Time Considerations

The synthesis of triphosphate prodrugs from nucleoside diphosphates and phosphorylating reagents can be accomplished in 1 day using microwave‐assisted reactions and 2 days using conventional heating. The synthesis of the pentafluorophenyl phosphorylating reagents from amino acid esters requires ∼1 day, whereas the synthesis of diphosphate nucleoside analogues is more laborious and may take up to 3‐4 days.

γ‐ProTriP prodrugs of adenosine are stable for several months when stored at –20°C under a nitrogen atmosphere.

### Author Contributions

**Camille Tisnerat**: Data curation; formal analysis; investigation; methodology; validation; visualization; writing—original draft; writing—review and editing. **Fabrizio Pertusati**: Conceptualization; formal analysis, investigation; methodology; resources; writing—review and editing. **Michaela Serpi**: Conceptualization; data curation; formal analysis; funding acquisition; investigation; methodology; project administration; resources; supervision; validation; visualization; writing—original draft; writing—review and editing.

### Conflict of Interest

The authors declare no conflict of interest.

## Data Availability

All data are provided in full in the Understanding Results section of this paper.
